# Canonical Sonic Hedgehog Signaling in Early Lung Development

**DOI:** 10.3390/jdb5010003

**Published:** 2017-03-13

**Authors:** Hugo Fernandes-Silva, Jorge Correia-Pinto, Rute Silva Moura

**Affiliations:** 1 Life and Health Sciences Research Institute (ICVS), School of Medicine, University of Minho, 4710‑057 Braga, Portugal; hugosilva@med.uminho.pt (H.F.-S.); jcp@med.uminho.pt (J.C.-P.); 2 ICVS/3B’s-PT Government Associate Laboratory, 4710‑057 Braga/Guimarães, Portugal; 3 Department of Pediatric Surgery, Hospital de Braga, 4710‑243 Braga, Portugal; 4 Biology Department, School of Sciences, University of Minho, 4710‑057 Braga, Portugal

**Keywords:** Sonic Hedgehog (SHH), lung development, endoderm specification, branching morphogenesis, signaling, transcriptional regulation

## Abstract

The canonical hedgehog (HH) signaling pathway is of major importance during embryonic development. HH is a key regulatory morphogen of numerous cellular processes, namely, cell growth and survival, differentiation, migration, and tissue polarity. Overall, it is able to trigger tissue-specific responses that, ultimately, contribute to the formation of a fully functional organism. Of all three HH proteins, Sonic Hedgehog (SHH) plays an essential role during lung development. In fact, abnormal levels of this secreted protein lead to severe foregut defects and lung hypoplasia. Canonical SHH signal transduction relies on the presence of transmembrane receptors, such as Patched1 and Smoothened, accessory proteins, as Hedgehog-interacting protein 1, and intracellular effector proteins, like GLI transcription factors. Altogether, this complex signaling machinery contributes to conveying SHH response. Pulmonary morphogenesis is deeply dependent on SHH and on its molecular interactions with other signaling pathways. In this review, the role of SHH in early stages of lung development, specifically in lung specification, primary bud formation, and branching morphogenesis is thoroughly reviewed.

## 1. Introduction

The development of a fully functional organism is a result of a highly regulated process, orchestrated by several signaling pathways and countless interactions that ultimately compose an intricate molecular network. These signaling pathways sharply control pivotal cellular processes, such as cell survival, proliferation, differentiation, migration, and apoptosis, among others and, thus, contribute to the formation of organs and systems.

Hedgehog (HH) signaling participates and regulates key cellular processes, such as growth, self-renewal, cell survival, differentiation, migration, and tissue polarity, during embryonic development [1]. Furthermore, it is also involved in vertebrate body patterning and morphogenesis of several organs, such as the lung [2]. Due to its role in different steps of development, HH signaling activity must be rigorously controlled or, otherwise, abnormalities may appear. In fact, aberrant activation of HH pathway is associated with different types of developmental disorders and cancers in brain, gastrointestinal, lung, breast, and prostate, to name a few; actually, there is an active field of research that aims to find HH pathway inhibitors and study their potential impact on distinct illnesses [3,4]. In addition, HH is also linked with stem and progenitor cells in organ repair and homeostatic mechanisms [5]. Therefore, HH signaling is of major importance from early embryo development to adult tissue maintenance, requiring a very tight spatial and temporal regulation to ensure its correct function.

Lung development relies on continuous interactions between the epithelial and mesenchymal compartment, coordinated by numerous transcription and growth factors that, overall, contribute to the proper formation of the respiratory system [6]. This developmental process requires the precise activation of several signaling cascades, namely, FGF (fibroblast growth factor), TGFβ-BMP (transforming growth factor β-bone morphogenetic protein), WNT (Wingless-related integration site), HIPPO, RA (retinoic acid), NOTCH, and SHH (Sonic Hedgehog) (reviewed by [7,8]). In this review, we aim to provide a description of the current knowledge and relevance of the canonical Sonic Hedgehog signaling pathway during early stages of lung development.

## 2. Canonical Sonic Hedgehog Signaling Pathway: Overview

HH gene was discovered in 1980 by Nusslein-Volhard and Wieschaus during a screening for mutations that altered fruit fly (*Drosophila melanogaster*) body plan [9]. In their studies, the authors showed the importance of this gene in *Drosophila* dorsal-ventral patterning and segmentation process. In vertebrates, there are three homologs of *Drosophila* segment polarity gene: desert hedgehog (*dhh*), indian hedgehog (*ihh*) and sonic hedgehog (*shh*) [10]. s*hh* is the most widely expressed HH gene and it is implicated in the developmental mechanisms underlying the formation of many organs, including the lung. On the other hand, *dhh* and *ihh* have more specific roles: *ihh* is associated with pancreas and bone development [11,12] whereas *dhh* is associated with male fertility, particularly spermatogenesis and testis organogenesis, and with peripheral nerve formation [13,14,15].

In mammals, SHH functional protein results from a precursor that undergoes a series of post-translational modifications. The process starts with an autoproteolytic step [16,17], that gives rise to a 25 kDa C-terminal peptide and a 19 kDa N-terminal product responsible for the signaling activity. Then, the N-terminal is modified by covalent addition of a hydrophobic cholesterol molecule [18,19] and by palmitoylation. These alterations contribute to SHH solubility and, consequently, to its long-range signaling capacity, as well as to the ability to form tissue gradients [20]. HH lipid modifications and their properties are extensively reviewed in [21]. After post-translation modifications, the functional protein is transported outside the cell with the assistance of a transporter membrane protein named Dispatched1 (DISP1) [22].

**Figure 1:**
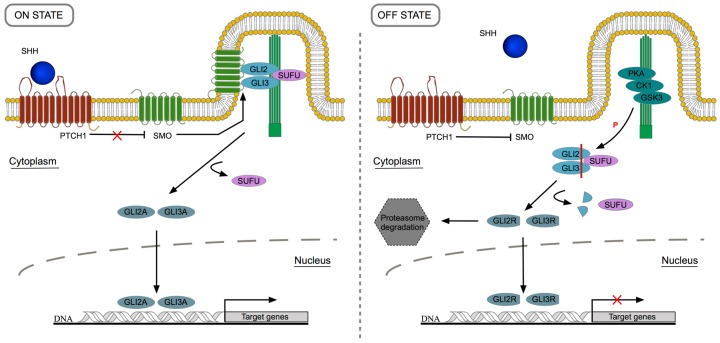
Sonic hedgehog signaling pathway. (1) ON state: SHH protein binds to PTCH1 receptor, at the cell surface level. This event abolishes SMO inhibition that can move to the primary cilia to induce GLI activation through SUFU detachment. Then, GLI2A and GLI3A translocate to the nucleus in order to promote the transcription of target genes; GLI2A acts as the main activator. (2) OFF state: In the absence of SHH ligand, PTCH1 inhibits SMO, and GLIs are phosphorylated by PKA/CK1/GSK3 complex. GLI2R and GLI3R are formed and can follow two possible destinations: proteasome degradation or translocation into the nucleus to repress the transcription of targets genes. In this case, GLI3R is the major repressor.

SHH may act in both autocrine and paracrine way, however, classically it is generally associated with paracrine signaling. In the canonical signaling pathway, SHH glycoprotein reaches the target cell and it is handled by the 12-transmembrane protein Patched1 (PTCH1). Normally, PTCH1 is responsible for the inhibition of the G-protein-coupled 7-transmembrane spanning protein Smoothened (SMO) [23]. When the signaling pathway is “ON”, SHH binds to PTCH1 and releases SMO inhibition that can move to the primary cilia, an essential cellular structure for the transduction of HH signal in vertebrates that acts as a SHH sensor [24,25]. Due to this event, zinc finger glioma-associated transcription factors (GLI) (*cubitus interruptus* family, in *Drosophila*) are transformed into their activator forms and dissociate from suppressor of fused (SUFU), a protein encoded by a tumor suppressor gene [26]. The activator form of GLIs, the final effectors of the pathway, are able to move to the nucleus and positively influence the transcription of specific target genes [27]. In the absence of SHH ligand, “OFF” state, PTCH1 inhibits SMO; GLIs are phosphorylated through PKA/CK1/GSK3 (cAMP-dependent protein kinase/casein kinase 1/glycogen synthase kinase 3) complex and undergo a proteolytic cleavage that allows the detachment from SUFU. As a consequence, GLIs are converted in their repressor form and subsequently transfer to the nucleus to repress the expression of particular target genes [28,29]. In Figure 1, the major events underlying SHH signaling are illustrated. Sonic Hedgehog machinery and transduction of signal are extensively reviewed in [30].

In vertebrates, there are three types of GLI transcription factors, GLI1, GLI2 and GLI3 that may be posttranslationally converted into their activator or repressor forms. In the presence of SHH ligand, GLI activator form is produced; among the three types, GLI2A is considered to be the primary pathway activator. On the other hand, in the absence of SHH ligand, GLI is truncated and converted in the repressor form. In this context, GLI3R is the main repressor and can translocate to the nucleus to suppress the expression of specific genes [31]. Basically, SHH signaling pathway modulates the balance between the activator and repressor forms of GLI, mainly GLI2 and GLI3, and, as result, GLI family members are able to regulate the expression of SHH target genes.

In addition to the abovementioned components of HH signaling pathway, there are other players such as PTCH2, cell surface protein growth arrest specific (GAS), cell adhesion CAM regulated by oncogenes (CDO) and brother of CDO (BOC) that function as co-receptors, and positively influence the pathway activity [32].

Regarding SHH signaling targets, they include pathway components as, for instance, *gli1* which acts only as a transcriptional activator through a positive feedback loop and does not contribute for HH signaling transduction [33]; furthermore, *ptch1* and *hip1* (that codes for the hedgehog-interacting protein, HIP1), are also downstream targets of SHH signaling but, in this case, they act by negative feedback [34,35]. In the particular case of membrane-bound protein HIP1, it recruits extracellular SHH and, hence, prevents its binding to the transmembrane receptor PTCH1 thus limiting SHH diffusion and signaling. This negative feedback loop explains why very high levels of SHH ligand lead to pathway repression instead of its activation [36]. Additionally, SHH signaling induces the expression of different sets of target genes, in a tissue-specific manner, as for example: secreted signaling proteins like BPM4 [37], cell cycle genes like N-Myc [38], and transcription factors such as *foxa2* (*forkhead box A2*, also known as *hnf-3β*, *hepatocyte nuclear factor-3β*) [39].

The HH canonical signaling pathway is, by far, the most studied. Nonetheless, there is increasing evidence that points to the existence of a non-canonical HH signaling. In this case, HH cellular response does not rely on the activation of GLI transcription factors, or HH signaling components interact with other molecular pathways (reviewed by [40,41]).

## 3. Lung Development: A Complex Process

The formation of the lung depends on complex interactions between different molecular factors during embryonic development. As a result, a well-designed organ able to perform the respiratory function and gas exchange through an efficient air-blood interface is formed. The respiratory system is composed of two main distinct tissues: the epithelial compartment that arises from the endoderm layer, and the mesenchymal compartment that has its origin in the mesoderm layer. During lung development, epithelium-mesenchyme interactions are constant and the intricate molecular network underlying this process depends on numerous signaling cascades. This process begins with the specification of lung cell fate in the ventral region of the foregut endoderm. Subsequently, these cells differentiate and proliferate, resulting in the formation of a laryngotracheal groove. From this structure, trachea emerges proximally and, in the distal region, ventrolateral buds start to sprout, and will later form the primary lung bronchi. Concurrently, the separation of two tubular structures leaning against each other occurs giving rise to the trachea and the esophagus. Finally, the mature lung structure results from successive branching of lung epithelium, by repetitive splitting and growth into the adjacent splanchnic mesenchyme, to form the airway tree [42]. The full maturation of the respiratory tract extends through post-natal life with the development of alveoli.

Lung development can be divided into five different stages, organized according to major events throughout gestation and that are common to both mice and humans: embryonic (embryonic day (E)9.5–12.5/mouse; 0–7 weeks/human), pseudoglandular (E12.5–16.5; 7–17 weeks), canalicular (E16.5–17.5; 17–27 weeks), saccular (E17.5 to Postnatal (P) 5; 28–36 weeks) and alveolar (P5–P30; 36 weeks to two years) [43,44]. The maturation of the human lung extends through postnatal life until the beginning of adulthood [45]. The first two developmental stages (early stages) are associated with the establishment of the conducting airways, and the last three (late stages) are linked with vascular development, alveolar development and with the reduction of mesenchymal tissue, that is crucial for the formation of the thin air-blood interface indispensable to gas exchange. The following sections will be devoted to exploring the importance of the Sonic Hedgehog signaling pathway during early stages of lung development.

### 3.1. Lung Endoderm Specification

The lung, thyroid, liver, and pancreas arise from the anterior-ventral region of the primitive foregut. This epithelial tubular structure, endoderm-derived, will differentially express tissue-specific genes along its anterior-posterior axis, which will determine the region where the future organs will emerge. Around E9.0 in the mouse, and day 28 in humans, it is possible to identify the expression of the homeodomain tissue-specific gene *nkx2.1* in the endoderm cells of the anterior foregut ventral region. This transcription factor, at this stage, specifies the foregut domain corresponding to the lung and thyroid, and that is why it is also known as thyroid transcription factor 1 (*ttf1*) or as thyroid-specific enhancer-binding protein (*t*/*ebp*) [46,47]. Additionally, at this gestational age, endoderm expresses *foxa2* that is required for primitive foregut tube closure [48], and *gata6* (a member of the zinc-finger family of transcription factors). The exact mechanism underlying the induction of lung cell endoderm specification, in both time and space, is still unknown. Even so, it is clear that numerous molecular players transduce signals between neighboring tissues and contribute to the initiation of this process. The respiratory lineage is determined by endodermal cells expressing *nkx2.1* [49]. Both *foxa2* and *gata6* cooperate with *nkx2.1* and contribute to the differentiation of primitive foregut endoderm into respiratory epithelial cell lineages [50,51]. Canonical WNT2/2b ligands, present in the adjacent lateral plate mesoderm, induce the expression of *nkx2.1* in the foregut endoderm [52]. Moreover, BMPs contribute to restricting its expression to the site of lung bud initiation [53]. In addition, FGFs are also thought to be implicated in foregut specification and seem to work in a concentration-dependent manner [54].

During lung specification, HH components, such as *shh*, are expressed in the ventral foregut endoderm and tracheal diverticulum [55]. On the other hand, *gli1*, *gli2*, and *gli3* are expressed in the splanchnic lateral plate mesoderm [56,57]. This pattern of expression is a clear illustration of SHH paracrine signaling, and it also indicates that epithelial-mesenchymal interactions occur as early as foregut specification. Recently, a complex molecular network that involves RA-SHH-BMP-WNT has been identified as indispensable for lung specification [58]. Basically, SHH endoderm expression is induced by RA produced in the neighboring mesoderm. Then, SHH signals back to the mesoderm in order to activate GLI2/3 transcription factors that, consequently, stimulate the expression of WNT2/2b ligands and BMP4. RA acts upstream SHH which, in its turn, acts upstream canonical WNT2/2b and BMPs that then induce *nkx2.1* expression and, consequently, lung specification programs. This signaling cascade is conserved in mice, *Xenopus*, and humans [58].

**Figure 2:**
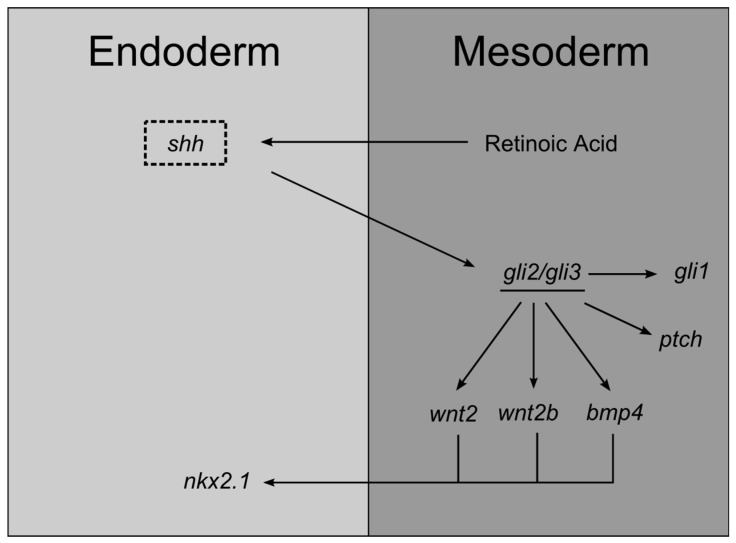
Schematic representation of SHH main interactions during lung endoderm specification.

Knockout mice studies confirmed the importance of SHH during foregut development and lung specification. *shh*-null mutants display a series of characteristics comparable to foregut defects observed in humans, namely, esophageal atresia/stenosis, tracheoesophageal fistula, and tracheal and lung anomalies; these malformations are so severe that these animals perish at birth. In these mutants, the tracheoesophageal septum fails to develop, causing a juxtaposition of the two tubes, which means that SHH is crucial for proper development of the esophagus, trachea, and lung. Likewise, this mouse mutant exhibits lower levels of *ptch* and *gli* in the surrounding mesoderm [55]. In fact, *gli2^−/−^* and *gli3^−/−^* mutants exhibit different types of foregut abnormalities that overlap with the defects observed in *shh^−/−^* mutants [57,59]. These findings suggest that SHH response is conveyed, at least in part, by GLI transcription factors. Curiously, *gli2^–/–^ gli3^–/–^* double mutants display an even more severe phenotype since they do not form trachea, lung, and esophagus, which suggests the involvement of other hedgehog family molecules, or that Gli2 and Gli3 have additional hedgehog-independent functions [59]. On the other hand, *gli1*-null mutants are apparently normal and viable [60]. In Figure 2, the main interactions of SHH signaling underlying endoderm specification are represented.

### 3.2. Branching Morphogenesis

#### 3.2.1. Primary Bud Formation

After the respiratory lineage has been determined, respiratory progenitors keep on proliferating and begin to differentiate, at different rates, to give rise to the unique cells that compose the lung. As a result, the laryngotracheal groove appears with the subsequent formation of the tracheoesophageal septum that will separate the esophagus from the trachea. Concomitantly, this new tube will grow distally and, eventually, primary lung buds will emerge. In mice, the sprouting of the ventrolateral buds is visible around E9.5, as a result of intricate epithelial-mesenchymal interactions and the activity of crucial transcriptions factors.

SHH signaling pathway contributes for primary bud outgrowth. Undeniably, *shh* is present in the lung from E10.5 and until E16.5 (late pseudoglandular stage). *shh* is highly expressed in the distal tips of the lung bud epithelium and absent from the most proximal regions [61]. Regarding its cellular surface receptor, *ptch1*, it is detected at high levels in the mesenchyme surrounding the distal epithelium, in E11.5 mouse lung [62]. Moreover, *gli1-3* are also expressed in the mesoderm. The importance of SHH signaling in primary bud morphogenesis can be disclosed through the analysis of *shh* and *gli* mouse mutants. *shh^−/−^* mouse mutant displays, among other phenotypes, single-lobe hypoplastic lungs, however, proximodistal differentiation of airway epithelium is preserved [63]. Furthermore, *gli2*, *gli3*, and *hip1* knockouts also exhibit lobation defects [36,56,59]. Likewise, in *shh^−/−^* embryos, development of lung buds is delayed about half a day, only appearing at E10.5. These abnormalities might be a consequence of primary bud malformation which, subsequently, affects lung branching morphogenesis [59].

#### 3.2.2. Branching Morphogenesis

After primary bud formation, bronchi elongate caudally and the epithelium undergoes a series of successive divisions that will give rise to the airways of the respiratory tree in a process known as branching morphogenesis. Simultaneously, the vascular network is formed and mesenchymal cells differentiate to give rise to the cartilage tissue. Crosstalk between the two cellular compartments, a key feature of branching morphogenesis, relies on diverse signaling pathways to deliver the appropriate response.

During branching morphogenesis, SHH signal transduction seems to be cilia-dependent. The analysis of *talpid^3^* chicken mutants revealed smaller lungs, with fewer branches and surrounded by fibrotic mesenchymal tissue as early as E6 [64]. The absence of TALPID3 (a centrosomal protein) causes a loss both of motile and non-motile primary cilia [65,66] leading to the disruption of SHH signaling [67,68]. The abnormal lung morphology observed in these mutants is a consequence of a defective SHH signaling, resulting in defects in both the epithelium and mesenchyme, although the distal structures remain unaltered [64].

The FGF signaling pathway is a major regulator of branching morphogenesis. The importance of FGF signaling for the initiation of bud branching was determined through analysis of the *Drosophila* tracheal system [69] and it seems to be conserved in both mammalian [70] and avian lung [71]. There are several FGF ligands present in the embryonic lung, but FGF10 stands out as a positive growth factor that promotes epithelial cell proliferation and expansion of the respiratory system [72,73]. In fact, *fgf10^−/−^* mutants fail to develop lungs due to branching morphogenesis disruption and impairment of primary bud formation [74]. Furthermore, FGF10 cognate receptor, FGFR2b, is also critical for lung morphogenesis since mice deficient in this particular gene display defective lungs [75]. FGF signaling is a classic example of the epithelial-mesenchymal interactions underlying pulmonary branching. *fgf10* is expressed in the lung mesenchyme surrounding the distal-most epithelium; *fgfr2b* is mainly found in the epithelial compartment. The spatial localization of the *fgf10* is normally associated with its role to induce endoderm proliferation and bud outgrowth, by acting as a chemotactic factor, thus modulating the induction and the direction of the airway branching [70,71,76]. Likewise, FGF9 is essential for proper lung development as a key regulator of mesenchymal proliferation. Disruption of *fgf9* signaling yields pulmonary hypoplasia and decreased airway branching due to a clear reduction of lung mesenchymal tissue and, consequently, decreased mesenchymal *fgf10* expression at branching regions of the lung [77]. *fgf9* is expressed in the epithelial compartment from E10.5–12.5 and in the prospective visceral pleura (mesothelium) from E10.5 onwards, and it signals to the mesenchymal compartment thereby contributing to the epithelium-mesenchyme interaction that controls lung branching [77].

The molecular mechanisms underlying lung branching must be finely regulated or otherwise, abnormalities will occur. In fact, *fgf10* expression levels are modulated by SHH signaling. *shh* is highly expressed in the distal epithelium of the growing buds, adjacent to the normal *fgf10* expression in the bud mesenchyme, whereas *hip1*, *ptch1*, *smo*, and *gli* are expressed in the mesenchymal compartment [36,45,57,62,63,78]. Despite the high levels of ligand in the distal bud tip, SHH signaling is not active due to the presence of HIP1, a SHH cell surface receptor, that binds to SHH and prevents signaling activation. As a consequence, *fgf10* expression is allowed in the growing mesenchymal tip. In the interbud/non-branching regions, SHH signaling is active and represses *fgf10* expression. Overall, this mechanism limits FGF10 signaling to active branching zones and controls lung bud outgrowth [70,79]. Actually, *hip1^−/−^* mouse mutants develop primary buds, nonetheless, the initial secondary branching fails to occur with an impairment in the lobulation pattern; these mice exhibit smaller lungs with a stunted airway tree [36]. In the absence of HIP1 (a SHH negative regulator), SHH signaling is enhanced and *fgf10* expression is almost completely repressed and, consequently, the initiation and growth of new secondary branches do not occur. Conversely, *fgfr2b* expression is not HIP1-dependent [36]. Additionally, overexpression of SHH in lung endoderm impairs *fgf10* expression [70]. Curiously, *hip1^−/−^*; *ptch1^+/−^* double mutants display a more severe phenotype (smaller lungs and thickened mesenchyme) than *hip1^−/−^* [36]. Indeed, *ptch1* overexpression in *hip1^−/−^* lungs partially rescued the lung phenotype (slight increase in lung size and lobation improvement). These findings suggest an overlapping role for these two receptors during lung branching [36]. On the other hand, *shh^−/−^* mutants display an increase in *fgf10* levels and, importantly, a widespread expression pattern which suggests that SHH is not necessary for *fgf10* expression but it is crucial to spatially restrict *fgf10* expression domain to the distal mesenchyme [63]. Furthermore, it has been shown that FGF10–FGFR2b signaling positively regulates *shh* epithelial expression [80]. The SHH-FGF10 feedback loop is highly complex since FGF10 and SHH regulate each other’s expression on the transcript level. These interactions seem to be mediated by ETV transcription factors (PEA3 group ETS domain transcription factor) that modulate *shh* expression. The FGF-ETV-SHH regulatory axis appears to be involved in controlling branching periodicity [81]. As a conclusion, SHH-FGF regulatory axis confines *fgf10* expression to active branching zones. It is worth mentioning that the expression pattern of SHH signaling members is quite conserved in the avian lung except for the high expression levels in distal tips. As a matter of fact, *shh* and its signaling machinery is completely absent from the distal tips. Indeed, in this region, SHH signaling is not active and *fgf10* is expressed in the mesenchyme, as it occurs in the mammalian lung [78]. Moreover, in vitro and in vivo studies have demonstrated that epithelial FGF9 induces/maintains *shh* expression contributing to the expansion and proliferation of subepithelial mesenchyme at early stages of branching [82].

In addition, WNT and BMP signaling are crucial for proper lung branching and are also downstream targets of SHH signaling. BMP4, a TGF-β superfamily member, is expressed in the distal bud epithelium and in the bordering mesenchyme; it inhibits epithelial cell proliferation and induces cell death in the mesenchyme [83]. *shh* mouse mutants, at E11.5, exhibit lower expression levels of *bmp4* in the mesenchymal compartment while in the epithelium remain unaltered [52]. Regarding canonical WNT signaling, there are many WNT ligands involved in branching morphogenesis. For instance, *wnt2* and *wnt7b* are expressed in the mesenchymal and epithelial compartment, respectively [63,84]. In *shh^−/−^* mutants, *wnt7b* expression levels are similar to wild-type lungs, while *wnt2* expression is downregulated [63]. Taken together, this evidence implies that SHH signaling primarily acts in pulmonary mesenchyme by modifying the expression levels of key signaling molecules. Non-canonical WNT signaling is also important for branching morphogenesis, namely *wnt5a* [85,86]; in fact, it has been demonstrated that lung-specific overexpression of *wnt5a*, in both mouse (E12-13) and chick (E10), causes a decrease in *shh* expression levels [86,87].

In the branching lung, SHH regulates the expression of transcription factors such as *foxf1* (that belong to the forkhead family of transcription factors) or T-box family of transcription factors (*tbx*). *foxf1*-null mice are lethal at very early stages of embryonic development; *foxf1* heterozygotes display a phenotype similar to *shh*-null mutants, namely respiratory failure due to lung hypoplasia and impairment in lung maturation, and foregut malformations [88]. In fact, *foxf1* expression is absent from lung tissue in *shh^−/−^* mutants. *foxf1* mRNA is present in the subepithelial mesenchyme regions of the airways, with higher expression levels in the mesenchyme surrounding the distal epithelium of the buds. This spatial distribution, complementary to *shh* expression pattern, favors a putative interaction between the two factors; actually, exogenous SHH is able to activate *foxf1* transcription in the developing lung [88]. Interestingly, in *shh^−/−^*; *gli3^−/−^* lungs *foxf1* expression levels are upregulated. Considering that the absence of SHH signaling causes an increase in Gli3R levels, it is likely that Gli3R contributes to the repression of *foxf1* in *shh^−/−^* lung mesenchyme and to growth inhibition [89]. These findings suggest that *foxf1* promotes proliferation and that it is probably one of the effectors of the SHH mitogen effect on the mesenchymal compartment.

TBXs are also downstream targets of SHH signaling. *tbx* are expressed in the developing lung as early as E9.5: *tbx1* is present in the lung epithelium while *tbx2–5* are present in the mesenchymal compartment [90,91]. In particular, TBX4 seems to be crucial for lung branching initiation through the interaction with FGF10 [92]. Moreover, *tbx2* regulates lung growth by repressing cyclin-dependent kinase inhibitor genes (*cdkn1a,b*) that repress cell cycle. In fact, *tbx2*-deficient mice exhibit hypoplastic lungs and diminished branching morphogenesis [91]. *tbx2,3* have redundant functions controlling proliferation in the pulmonary mesenchyme and act upstream of WNT signaling but downstream of SHH signaling [93]. In fact, *shh^−/−^* and *smo^−/−^* lungs display reduced levels of *tbx2-3* corroborating this interaction [89,93]. Furthermore, in *smo*-deficient lungs, *axin2* expression levels (WNT target gene) are strongly reduced; nonetheless, *tbx2* mesenchymal re-expression reverts this scenario [93]. *tbx2,3* convey SHH signals during lung branching morphogenesis. SHH-*tbx2/3* molecular network contributes to the proliferative expansion of lung mesenchyme and branching morphogenesis by repressing WNT signaling antagonists, and by regulating the expression of cell-cycle regulating genes such as *cdkn* [93].

**Figure 3:**
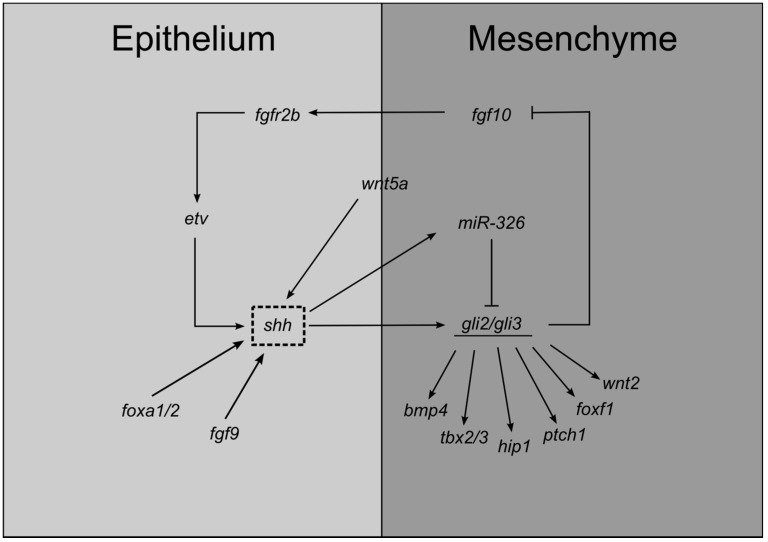
Schematic representation of SHH main interactions during lung endoderm specification.

On the other hand, *shh* is a target of *foxa1/2* transcription factors. Deletion of both *foxa1* and *foxa2* leads to an impairment on branching morphogenesis, as early as E12.5, which may be partially due a decrease/absence of *shh* [94]. The contrary is not true since *foxa1/2* are not downstream targets of SHH signaling [94]. Additionally, transcriptional regulation can be modulated by small, noncoding RNAs (microRNAs) that affect gene expression post-transcriptionally. Recently it has been demonstrated that miRNA-326 is not only a downstream target but also a negative modulator of SHH signaling by direct modification of *gli2* and *smo* [95]. In Figure 3, the principal interactions of SHH signaling in branching morphogenesis are indicated.

Additionally, in Table 1, we have summarized SHH upstream and downstream genes that contribute to early lung development.

## 4. Final Remarks

SHH is a key morphogen that regulates pulmonary morphogenesis by triggering tissue-specific responses that contribute to the formation of a complex respiratory structure. In this review, we have highlighted the major roles of the canonical SHH signaling pathway in early stages of lung development. Regardless of the stage, SHH contributes to the epithelial-mesenchymal interactions that underlie lung development. In this particular case, SHH produced by the epithelial compartment acts on the pulmonary mesenchyme by regulating the expression of key signaling molecules, such as FGF10 and BMP4, just to name a few. The SHH molecular network must be sharply controlled in both time and space or, otherwise, severe congenital defects will occur. Additionally, canonical SHH signaling is also involved in lung alveolarization and maturation, and it has been implicated in adult lung diseases, such as pulmonary fibrosis, asthma, and chronic obstructive pulmonary disease (revised in [96]). Moreover, signaling pathways that regulate developmental processes and organ homeostasis usually play critical roles in tumorigenesis. Abnormal hyperactivation of the Hedgehog signaling pathway has been described in different subtypes of lung cancer. For instance, in small-cell lung cancer, it has been reported an increase in SHH ligands or an overexpression of GLI proteins [97]. On the other hand, in lung cancer cell line A549 and in some lung tumors, the loss of HIP1 (SHH pathway antagonist) has been documented [98]. In all these cases, aberrant SHH signaling activation upregulates cancer cell proliferation, maintains cancer stem cells and, eventually in some cases, enhance their metastatic potential (reviewed in [99,100]).

Lung development is orchestrated by many signaling cascades perfectly coordinated to give rise to a fully functional organ. Perhaps, there are still unknown molecular interactions that need to be uncovered, and it is likely that SHH may be a player in these hypothetical networks.

## Figures and Tables

**Table 1:** Summary of SHH signaling upstream and downstream targets that contribute to early lung development.

Developmental Stage	Gene/Signaling Pathway	Upstream	Downstream	Molecular Interaction	Reference
Lung Specification	*bmp4*		×	Direct	[58]
	*gli1*		×	Direct	[55]
	*gli2*		×	Direct	[58,59]
	*gli3*		×	Direct	[55,58,59]
	*nkx2.1* (*ttf1* or *t/ebp*)		×	Indirect	[58]
	*ptch*		×	Direct	[58]
	RA pathway	×		Direct	[58]
	*wnt2*		×	Direct	[58]
	*wnt2b*		×	Direct	[58]
Branching Morphogenesis	*bmp4*		×	Direct	[55,63,83]
	*cdkn1a*		×	Indirect	[93]
	*cdkn1b*		×	Indirect	[93]
	*etv*	×		Direct	[81]
	*fgf9*	×		Direct	[82]
	*fgf10*	×	×	Direct	[63,70,79,80,81]
	*foxa1/2*	×		Direct	[94]
	*foxf1*		×	Direct	[88,89]
	*gli2*		×	Direct	[59]
	*gli3*		×	Direct	[59]
	*hip1*		×	Direct	[36]
	*miR-326*	×	×	Direct/indirect	[95]
	*ptch1*		×	Direct	[36]
	*tbx2*		×	Direct	[89,93]
	*tbx3*		×	Direct	[89,93]
	*wnt2*		×	Direct	[63]
	*wnt5a*	×		Direct	[85,86]
